# Cognitive ability, education and socioeconomic status in childhood and risk of post-stroke depression in later life: A systematic review and meta-analysis

**DOI:** 10.1371/journal.pone.0200525

**Published:** 2018-07-16

**Authors:** Ellen V. Backhouse, Caroline A. McHutchison, Vera Cvoro, Susan D. Shenkin, Joanna M. Wardlaw

**Affiliations:** 1 Centre for Clinical Brain Sciences, University of Edinburgh, Edinburgh, United Kingdom; 2 Centre for Cognitive Ageing and Cognitive Epidemiology, University of Edinburgh, Edinburgh, United Kingdom; 3 Scottish Imaging Network, A Platform for Scientific Excellence (SINAPSE), Scotland, United Kingdom; 4 Geriatric Medicine, Department of Clinical and Surgical Sciences, The University of Edinburgh, Edinburgh, United Kingdom; 5 UK Dementia Research Institute at The University of Edinburgh, Edinburgh Medical School, Edinburgh, United Kingdom; Charite Universitatsmedizin Berlin, GERMANY

## Abstract

**Background:**

Depression after stroke is common and is associated with poorer recovery. Risk factors such as gender, age and stroke severity are established, but it is unclear whether factors from earlier in life might also contribute.

**Methods:**

We searched MEDLINE, PsycINFO, EMBASE and meta-analysed all available evidence on childhood (premorbid) IQ, socioeconomic status (SES), education and stroke in adulthood. We included all studies reporting data on >50 patients, calculating overall odds ratios (OR), mean difference, correlation, 95% confidence intervals (CI) and 95% predictive intervals (PI) using random effects methods. We quality assessed all studies, performed sensitivity analyses, assessed heterogeneity and publication bias.

**Results:**

We identified 33 studies including 2,664 participants with post-stroke depression and 5,460 without (314 participants not classified). Low education (< = 8 years) was associated with post-stroke depression in studies which defined depression as score of mild and above on a depression rating scale (OR 1.47 95% CI 1.10–1.97, p<0.01) but not in studies where depression was defined as severe depressive symptoms or a clinical diagnosis of major depression (OR 1.04 95% CI 0.90–1.31, p = 0.60). Low education was not associated with an increased risk for post-stroke depression in studies that adjusted for age and sex (OR 0.86 95% CI 0.50–1.48 p = 0.58). Those with post-stroke depression had fewer years of education than those without post-stroke depression (MD 0.68 95% CI 0.05–1.31 p = 0.04). Few studies adjusted for vascular risk factors or stroke severity. Heterogeneity between studies was moderate and was partly explained by severity of depression. In the one study identified premorbid IQ did not differ between those with post-stroke depression (mean IQ 10.1.8 SD 9.8) vs those without (mean IQ 104 SD 10.1). There were no studies that examined childhood socioeconomic status and risk of post-stroke depression.

**Conclusions:**

Having less education is associated with an increased risk of post-stroke depressive symptoms but with large confidence intervals and heterogeneity. Future studies should explore the relationship between early and late life risk factors to improve risk identification and to target prevention and treatment strategies.

## Introduction

Stroke is the commonest cause of dependency in adults in the developed world [1] and also causes cognitive, physical and psychiatric disabilities. Depression is one of the most common neuropsychiatric disturbances following stroke, occurring in approximately 31% of patients during the first 5 years [2]. People with post-stroke depression experience greater impairment, including worse cognitive impairment, more substantial reductions in activities of daily living, and increased mortality [3] compared with non-depressed stroke patients. Post-stroke depression can severely impair physical rehabilitation and recovery [3].

Several risk factors for post-stroke depression have been proposed. These include gender, medical and psychiatric history [3, 4], age, and social support [4] as well as factors relating to the stroke such as severity and degree of resulting disability [3]. However, evidence supporting these factors is mixed and they only explain some of the variance in post-stroke depression. Factors from earlier in life may also be important. Two recent meta-analyses [5, 6] reported that low childhood cognitive ability, low childhood socioeconomic status (SES) and low education were associated with an increased risk of stroke and subclinical cerebrovascular disease on neuroimaging or at post mortem. Few studies have specifically examined the relationships between these childhood factors and post-stroke depression, but it is possible that an association exists via the relationships between early life factors and vascular disease. A previous review [7] of 10 studies found no association between education and post-stroke depression. However, this review only included papers published in English between 1995 and 2012 which directly analysed education as a risk factor, and did not perform a meta-analysis.

To address the question of whether childhood cognitive ability, SES or education affect the risk of post-stroke depression in later life, we performed a systematic review and meta-analysis of all published literature.

## Method

The methodology of this systematic review has been described previously [5, 6]. We used the PRISMA and MOOSE guidelines [8] (see S1 Table), and registered the protocol prospectively on Prospero (registration number: CRD42015016701).

Using a detailed search strategy (Appendix in S1 File) we searched PsycINFO (1806-present), MEDLINE (1966-present) and EMBASE (1980-present) for papers published until 6 April 2017 using OVID SP UI03.16.00.110. We also checked reference lists of identified papers and relevant review papers and hand searched the previous five years of *Stroke*, *Neurology* and *International Journal of Epidemiology*.

Each abstract and title were screened by one reviewer and all potentially relevant texts were independently screened by two researchers (EB or CM) for relevance. Disagreements regarding eligibility were resolved through discussion between authors.

We included studies that provided data on one or more early life factors (education, social class, IQ) in relation to a diagnosis of depression or measurement of depressive symptoms following stroke. We defined depressive symptoms as any measurement of mood using a valid scale conducted at any time following a stroke. Valid scales included the Beck’s Depression Inventory, the Montgomery Asberg Depression Rating Scale (MADRAS) and the Hamilton Depression Rating Scale (HDRS) (higher score indicates worse depression/depressive symptoms). A diagnosis of major depression according to classification systems such as the Diagnostic and Statistical Manual of Mental Disorders (DSM) was also included. We included general intelligence (IQ) measurements performed up to age 18 and estimates of premorbid IQ using valid tools (e.g. the National Adult Reading Test (NART)). All measures of childhood education were included (duration, attainment). We included childhood SES measures such as parental occupation or education.

We excluded papers with less than 50 patients, those focusing on a particular non-stroke patient population (e.g. Multiple Sclerosis), without primary data, not reporting data on humans aged 18 or over, or abstract only publications. We considered papers in any language. We used double data extraction conducted by two researchers.

We quality assessed the included studies on six potential sources of bias [9]: representativeness of the sample to the general population, whether study attrition was reported, how education and post-stroke depression were measured, whether results were adjusted for confounders and appropriateness of the statistical analysis. We rated each of these on a 4-point scale (corresponding to unclear, no, partly, yes) with a maximum score of 24. We counted each study only once, being careful to avoid double counting where more than one paper referred to the same study.

We standardised all education results to represent a reference level of high education, based on each paper’s categorisation of education in the majority of papers. Low education was defined as approximately 6–8 years (or less than high school) and high education as 9 years and above (or high school and above).

We used Review Manager V.5.3 to calculate overall odds ratios (OR) or mean differences (MD) and 95% confidence intervals using a random effects model. Where multiple statistics were reported, we used the one that maximised data available for meta-analysis. Where possible, we used the results adjusted for depression or vascular risk factors over crude results. Where necessary, we calculated odds ratios from frequency data and we analysed correlation coefficients using the package ‘metacor’ for R V.3.0.1. We analysed papers which reported means years of education in a separate group. We assessed heterogeneity using the I^2^ statistic and publication bias with funnel plots. We further calculated 95% prediction intervals which incorporates existing heterogeneity and quantifies the likely range of associations between education and depression in similar future studies [10].

We performed post-hoc sensitivity analyses on several clinically important subgroups and factors previously reported to be associated with post-stroke depression.

## Results

**Fig 1:**
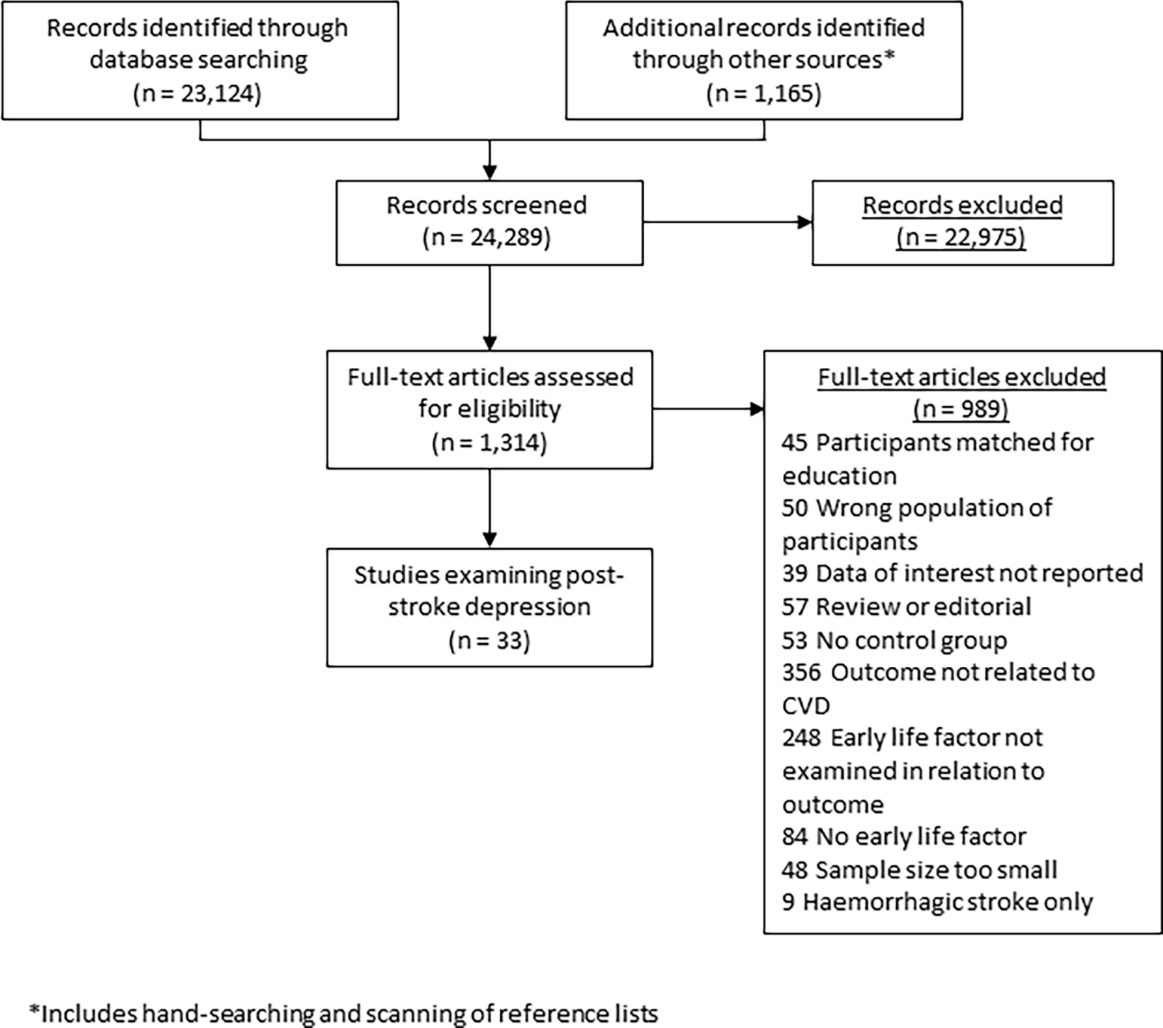
PRISMA flow chart of search process.

We identified 24,289 titles and abstracts after removal of duplicates (Fig 1), from which we identified 1,314 full text articles. The commonest reason for exclusion was no measurement of depression. 33 articles, all examining education and post stroke depression met inclusion criteria (see Table 1 for summary of included studies and Table A in S1 File for full details). One of these articles [11] also examined premorbid cognition. There were no studies examining childhood SES and post-stroke depression.

**Table 1:** Table of studies included in systematic review of early life factors and post-stroke depression.

	Childhood IQ	Education	Childhood SES
Number of papers identified	1	33	0
Number of studies included	1	33	0
**Study setting**			
Population	0	3	0
Hospital	1	22	0
Community	0	1	0
Outpatient clinic	0	7	0
Total number of participants	205	8,377	0
Total number of depressed patients	37^a^	2,664	0
Total number of non-depressed patients	98^a^	5,460	0
Age range of included studies	72–73	27–85	0
Quality score^b^	20	20 (1.5)	NA
Range	NA	18–23	NA

^a^ Data not reported for 2 studies.

^b^ Median (interquartile range).

SES: socioeconomic status.

### Quality assessment and publication bias

The quality of the included papers was good, with scores from 18-23/24 (median = 20). The main risk of bias was regarding sample representativeness (Figure A in S1 File).

There was no evidence of publication bias among papers examining education level and depression (Figure B in S1 File). Due to the small number of studies, it was not possible to assess publication bias in papers reporting mean years of education or correlation coefficients.

### Premorbid IQ

One paper [11] (n = 205) examined premorbid IQ using the National Adult Reading Test Revised (NART-R) and post-stroke depression diagnosed using The Diagnostic and Statistical Manual of Mental Disorders (DSM) criteria (37 post-stroke depression; 98 no depression). NART scores, transformed into an IQ score, were higher (better premorbid IQ) in those without depression (mean: 104.0, SD 10.1) compared to those with post-stroke depression (mean: 101.8, SD 9.8) but this difference was not statistically significant.

### Education

Thirty three studies [11–43] (n = 8,377, range: 64–1,068) examined education and post-stroke depression (2,664 post-stroke depression; 5,460 no depression participants, 314 participants not classified) aged 27–85 at follow up. Education level was assessed as duration (i.e. ≤8 years vs >8 years) in 12 studies [12, 17, 19, 21, 24, 27, 30, 32, 33, 33, 34, 36], attainment (i.e. <High School vs ≥High school) in 13 studies [13–16, 18–20, 22, 23, 28, 29, 31, 35, 35] and mean years of education in 8 studies [11, 37–43]. Most studies were conducted in Europe or North America (17 studies) however some were based in the Asia Pacific Region (12 studies), Africa (1 study), the Middle East (2 studies) and South America (1 study). Twenty three studies were based in hospitals, 6 were outpatient studies and 4 were population or community based studies.

#### Education level and depression

Of the 33 studies 8 papers [24–31] (n = 1785) reported ORs (711 with and 1074 without post-stroke depression) and 12 papers [12–23] (n = 3879) reported frequencies of presence of post-stroke depression by educational attainment or duration (1434 with and 2445 without post-stroke depression) which we used to calculate unadjusted ORs. Three of these studies [26, 27, 30] reported adjusted odds ratios.

Eleven studies [12,13,15,16,20,21–23,25,29,30] (n = 1,937: range = 91–329) defined post-stroke depression according to a cut off score indicating the presence of mild depressive symptoms or above. These were measured on rating scales including the Montgomery Asberg Depression Rating,Scale (MADRAS) (4 studies [15, 16, 20, 25]; score ≥7), the Hamilton Depression Rating Scale (HDRS) (2 studies [21, 23]; score ≥8 mild depressive symptoms), the Becks Depression Inventory (BDI) (2 studies [12, 13]; score ≥10), the Geriatric Depression scale (GDS) (2 studies; Short version [29]: score >5, short version ≥7[30]) and a Chinese self-report depression scale (1 study [22]).

Nine studies [14,17–19,24,26–28,31] (n = 3,754: range = 105–1,068) defined post-stroke depression as a clinical diagnosis of depression or moderate to severe depressive symptoms on a self-report scale. This included four studies [17–19, 24] which classified participants with depression by a diagnosis of major depressive disorder (MDD) according to DSM criteria. Two studies [28, 31] used the Centre for Epidemiologic Studies Depression scale (CES-D) which diagnoses a depressive episode using the DSM criteria. Three studies defined participants with moderate or severe depressive symptoms according to depression rating scales: the Hamilton Depression Rating Scale (HDRS) (score of ≥21, 1 study[27]); the Bengali version of the Geriatric Depression scale (GDS) (score of ≥21, 1 study[26]) and the Patient Health Questionnaire 8 (PHQ-8) (score ≥10, 1 study[14]).

**Fig 2:**
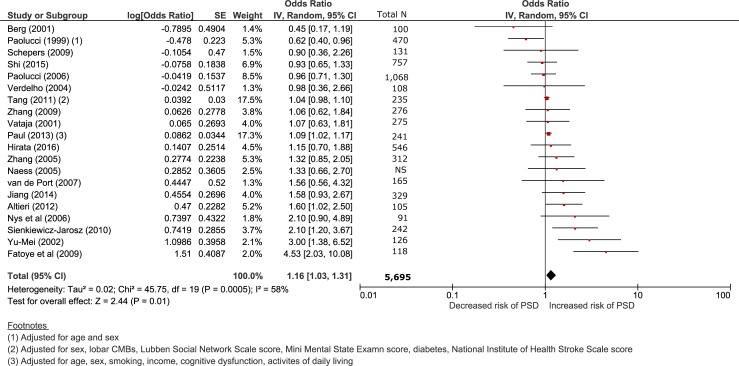
Forest plot comparing low vs high education and risk of depressive symptoms following stroke. OR<1: low education decreases risk post-stroke depression; OR>1 low education increases risk of post-stroke depression. Random effects model.

Overall low education (<9 years) was associated with increased risk of post-stroke depression or depressive symptoms (OR 1.16 95% CI 1.03–1.31, p = 0.01, Fig 2). Heterogeneity between studies was moderate (I^2^ 58%). The 95% prediction interval was 0.98 to 1.52.

**Sensitivity analysis**

**Fig 3:**
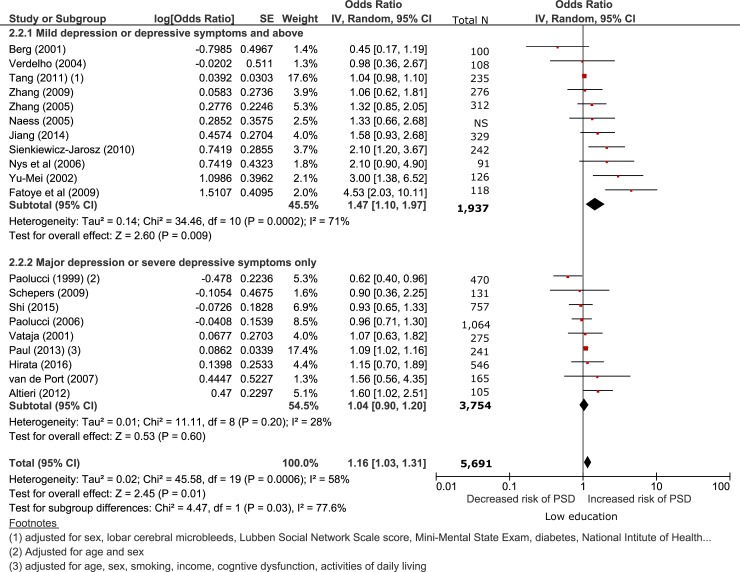
Sensitivity analysis comparing studies with depression defined as mild symptoms and above vs clinical depression or severe depressive symptoms only.

**Fig 4:**
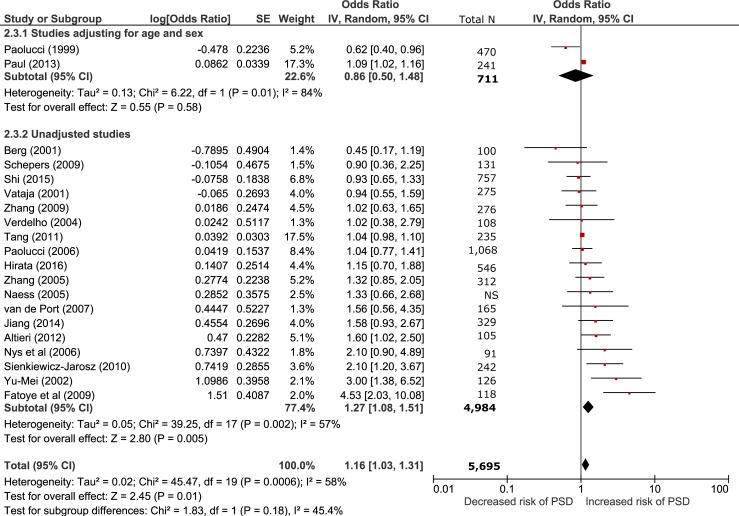
Sensitivity analysis comparing studies adjusted for age and sex vs unadjusted studies.

We conducted several post hoc sensitivity analyses examining education level and post-stroke depression (see Figs 3 and 4 and Figures C-J in S1 File.). Definition of post-stroke depression explained some of the between study heterogeneity (χ^2^ (1) = 4.47 p = 0.03). Low education was associated with increased risk of post-stroke depression defined as a score of mild and above on a depression rating scale (n = 1,937, OR 1.47 95% CI 1.10–1.97, p<0.01, 95% PI 0.47–3.00) but not major depression or severe depressive symptoms (n = 3,754 OR 1.04 95% CI 0.90–1.31, p = 0.60, 95% PI 0.69–1.51) (Fig 3).

Low education was associated with post-stroke depression in studies which did not adjust for age and sex (18 studies, n = 4,984; OR 1.27 95% CI 1.08–1.51, p<0.01, 95% PI 0.95–2.14) but not in studies which did adjust for age and sex (2 studies, n = 711; OR 0.86 95% CI 0.50–1.48 p = 0.58, 95% PI 0.96–1.81) (Fig 4).

The risk of post-stroke depression did not differ according to the depression scale used, participants age (<65 vs ≥65 years), first stroke only vs recurrent or unspecified stroke, past history of depression as an exclusion criteria (yes vs no), time since stroke (≤6 months vs >6 months), study setting (hospital or outpatient clinic vs population-based) and country of origin (Europe or North America vs Asia Pacific region or Africa).

Exclusion of the paper [23] with the lowest quality score, or the two studies [20, 25] with unclear definitions of education levels, did not significantly alter the results (OR 1.23 95% CI 1.02–1.49, p = 0.03; OR1.24, 95% CI 1.03–1.50, p = 0.02).

#### Mean years of education and depression

**Fig 5:**
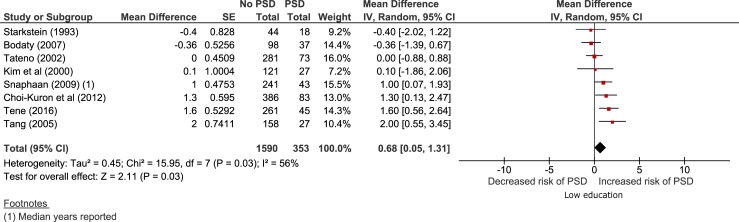
Mean years of education for those with and without post-stroke depression. Random effects model for the mean difference. Negative mean difference = lower education decreases risk of post-stroke depression and positive mean difference = higher education decreases risk of post-stroke depression.

Of the 33 studies, 7 [11, 37, 38, 40–43] (n = 1943: range = 80–469) reported mean years of education for participants with and without post-stroke depression (273 post-stroke depression and 1251 no post-stroke depression) and one study [39] reported median years of education. Post-stroke depression was diagnosed using the DSM criteria (5 studies[11, 38, 40–42]), and the BDI [37], the GDS [43] and the Hospital Anxiety and Depression Scale (HADS) [39] in one study each. Participants with post-stroke depression had significantly fewer years of education than those without post-stroke depression (MD 0.68 95% CI 0.05–1.31 p = 0.03, Fig 5). None of these papers adjusted for vascular risk factors.

Heterogeneity between studies was moderate (I^2^ 56%). The 95% prediction interval was 1.14 to 2.50. There were too few studies to conduct any sensitivity analyses.

#### Correlation between education and depressive symptoms

Of the 33 studies, 5 [32–36] (n = 831: range = 64–300) reported correlation coefficients for education and post-stroke depressive symptoms at age 56–70. Most studies used years of education while one [35] used educational attainment ranging from 1 (Primary school) to 7 (University degree). One study [35] provided risk factor adjusted results. Depressive symptoms were measured using the HADS in two studies [32, 36], the HDRS [34], the GDS [33]and the CES-D [35] in one study each.

**Fig 6:**
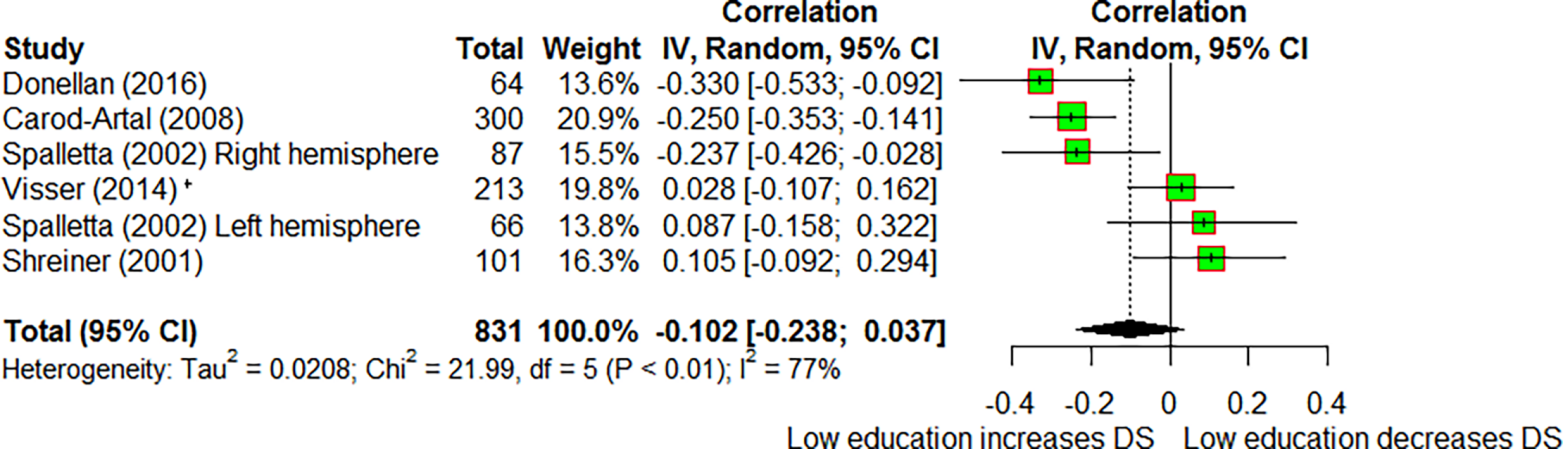
Forest plot showing correlation between education and depressive symptoms in stroke patients. Negative correlation = low education increases depressive symptoms; Positive correlation = low education decreases depressive symptoms. DS = depressive symptoms. * adjusted for sex, income, smoking, age, cognitive dysfunction and activities of daily living. DS = depressive symptoms.

Overall correlation between education and depressive symptoms did not reach statistical significance (r = -0.10 95% CI -0.24–0.04, p = 0.15, Fig 6), although the effect was in the same direction as the effect of the alternative education measures above on post-stroke depression. Heterogeneity was high between studies (I^2^ 77.3%) but the data were too sparse for sensitivity analyses.

## Discussion

Our meta-analysis is the first comprehensive examination of all data on education and risk of post-stroke depression and suggests that longer duration of education is associated with a decreased risk of depression following stroke occurring in later life. Less versus more education was associated with a 16% relative increase in post-stroke depression. This relative risk translates to an absolute increase in post-stroke depression risk of approximately 5.9/1,000 for lower versus higher education but with wide confidence intervals and heterogeneity. Furthermore, participants with post-stroke depression had an average of 0.68 fewer years of education than those without post-stroke depression. These findings suggest important implications for predicting risk of post-depression and of recovery after stroke. A previous review [7] reported significant associations between education and depression in only two of the studies they identified, but only included 10 papers (versus the 33 included here) and excluded studies which did not analyse education directly, which reported mean years or correlation coefficients, and did not include a comprehensive meta-analysis such as performed here.

Our sensitivity analysis showed that low education was associated with increased risk of post-stroke depressive symptoms, defined as mild symptoms and above, but not severe depressive symptoms or a clinical diagnosis of depression. Studies using a cut of score of mild depressive symptoms to define post-stroke depression also included participants with moderate to severe depressive symptoms. Therefore it is unclear whether the association between education and post-stroke depression is stronger for milder depressive symptoms or whether these differences are due to methodological differences between studies. The association between years of education and depression severity was examined in only one study [15], which found no association. However the number of participants with major depression in this study was low (n = 14) and so this should be examined in future studies with larger sample sizes.

The diagnosis of depression in stroke populations is more difficult than in those without stroke and many depression scales were not originally developed for patients with stroke. Stroke patients may suffer from symptoms such as fatigue and lack of appetite after stroke, which may lead to inflated scores on depression scales containing a somatic component (e.g. BDI, HDRS) compared to those that do not include such items (e.g. HADS, MADRAS). No studies adjusted for other common associates of post-stroke depression such as fatigue, although these factors should be considered when conducting research into post-stroke depression. Meta-analysis [44] suggests that the CES-D, HDRS and PHQ-9 are the most promising options to screen for post-stroke depression. However in our review CES-D and the HDRS were only used in three [28, 31, 35], four [21, 23, 27] studies respectively. One study [14] used the PHQ-8 which contains one less question that the PHQ-9.

We found considerable heterogeneity between studies and calculated prediction intervals in addition to confidence intervals. The prediction interval is useful in the presence of heterogeneity as it provides a range of effects that would be expected from a new study with similar characteristics to the current studies [10]. The 95% prediction interval indicates that although low education on average is associated with an increased risk of post-stroke depression, some future studies may not find an association. Specifically in a future study the expected association between education and post-stroke depression would be between 0.98 and 1.52 with 95% confidence.

Confounders were poorly addressed, either because authors reported unadjusted results or because frequency data were used to calculate unadjusted odds ratios. In those papers that did include adjustment for confounders (four studies), there was variation in the number and type used. Of particular importance are sex and age which were adjusted for in four and three studies respectively. Female sex is a risk factor for post-stroke depression [45] and in the cohorts included in this review females may be more likely to have lower levels of education. Similarly older participants may have less education and may be more vulnerable to depression due to more advanced vascular disease. Our sensitivity analysis showed that low education was associated with post-stroke depression in studies which did not adjust for age or sex but there was no association in studies which did adjust for age and sex, however this included only two studies. Inclusion of the one study [41] that did not adjust for age but did adjust for sex did not alter the results of the sensitivity analysis. Other confounders which were adjusted for include stroke severity (one study) and impairments in activities of daily living (two studies). More studies are needed to examine associations between education and post-stroke depression after adjustment for risk factors for post-stroke depression, particularly age and sex.

No studies adjusted for other early life factors or adult SES. It is likely that education is interrelated with premorbid IQ and that all factors are associated with an increased risk of stroke, but it was not possible to assess the independence of these early life risk factors on post-stroke depression from the current literature as the only paper which examined both premorbid IQ and education reported mean values for each separately. Education is also strongly associated with adult SES which is itself a risk factor for stroke and depression [46]. Previous research has suggested that education and measures of SES such as income may have independent associations with health outcomes [47]. However future studies should examine associations between education and post-stroke depression when controlling for adult SES.

While methodological differences between studies might also contribute to heterogeneity, we were not able to find any evidence that the type of depression scale used, whether set in hospital in-patient, out-patient or population-based, world region, inclusion or exclusion of prior stroke or prior history of depression or patient age accounted for the heterogeneity. Other differences between studies include the interval between stroke and post-stroke assessment which varied from within 48 hours to greater than 18 months. However, prevalence of depression after stroke has been found to be stable across studies conducted at different time points[48, 49] and our sensitivity analysis showed that the effect of education and depression did not differ between before and after 6 months post stroke.

Exclusion criteria varied among studies. Seven studies excluded participants with a pre-stroke history of depression and eight studies excluded participants with a previous history of stroke, both factors which have been identified as risk factors for post-stroke depression[7]. Our sensitivity analyses showed that studies including participants with a history of stroke or depression reported a higher effect of education on post-stroke depression risk than those that excluded such participants, but these differences were not significant. No studies adjusted for history of depression or stroke in their analysis.

The majority of the identified studies were cross sectional. Depressive symptoms may fluctuate over time and a single measurement at a relatively arbitrary point in time may only provide a snapshot of symptoms, particularly mild symptoms. A more comprehensive measure of depressive symptoms at multiple time points may offer a more precise estimate of the association between education and post-stroke depression. Although 11 of the identified studies were longitudinal only 5 reported measures of depression or depressive symptoms at multiple time points in relation to education. In two studies [37,24] low education level was associated with depression measured at baseline, 1 month [24] and 3 months [37]. Another study reported that those whose depressive symptoms worsened between baseline and 6 month follow up were less educated than those whose symptoms stayed the same [43]. However two studies [18,20] found no association between education level and depressive symptoms measured at baseline, 6 months, 12 months [18] and 36 months [20] post-stroke.

The majority of studies were from Europe or North America followed by the Asia Pacific Region. Our sensitivity analysis found no difference between studies conducted in Europe or North America compared to the Asia Pacific Region or Africa. However, we were only able to conduct sensitivity analysis on a subset of papers and social and educational disparities may vary between these world regions.

We excluded studies with less than 50 participants as results from small studies can be less reliable[50]. Therefore, although our funnel plot showed no publication bias, we may have excluded smaller non-significant studies.

### Strengths and limitations of the review

Our systematic review had limitations. Resources prevented contacting authors for original data where information on education may have been collected but not reported. The sensitivity analysis was not able to account for all the heterogeneity.

Strengths of the review include a pre-specified published protocol, validated search strategy, double data extraction. We followed published guidelines and used exemplary methods on conduct of systematic reviews and meta-analyses, and established scale for quality assessment which showed an overall high level of study quality. Some sample sizes were small, however there was a reasonable total sample size for many of the analyses producing a comprehensive literature review and meta-analyses amassing data on 8,377 participants. Some analyses lacked power and we may have missed some significant associations through lack of significant data.

### Implications and conclusions

The aetiology of post stroke depression is still unclear. Some researchers propose that the primary mechanism linking stroke and depression is biological in which brain damage caused by ischaemic lesions disrupt neural circuits involved in mood regulation. Others propose that depression is caused by dysfunctional psychosocial adjustment following the stroke. It is likely that post-stroke depression is of multifactorial origin and a combination of biological and psychosocial mechanisms.

There are several possible explanations for the observed relationship between education and post-stroke depression. Our previous systematic reviews [5, 6] showed that low education was associated with an increased risk of stroke and subclinical cerebrovascular disease (e.g. white matter hyperintensities). It may be that low education leads to more severe stroke which in turn increases depression. Alternatively, low education may increase imaging markers of vascular disease which increase the risk of depression and stroke since a relationship between WMH and depression is suggested [51].

Our findings show an association between lower educational attainment and increased risk of depression following stroke. However further studies are needed to confirm this and our findings should be interpreted with caution due to the substantial heterogeneity between studies, the relatively large confidence intervals around the effect sizes and the fact that many studies did not adjust for potential confounders. Health disparities have been widely discussed and emphasise the importance of addressing social inequality to improve health outcomes. Post-stroke depression is often considered a treatable complication of stroke, but antidepressants are only partially effective and, despite its high prevalence, it remains poorly recognised and undertreated and trials are ongoing. Identifying additional aspects of the mechanisms of post-stroke depression and modifiable risk factors may lead to more specific therapeutic interventions to target those most at risk and may help design future policy. Future research should examine the combined effect of education and other early life factors on post-stroke depression after adjusting for possible confounders.

## Supporting information

S1 Table PRISMA checklist. (DOCX)

S1 File All supporting information. (DOCX)

S2 File Data. (XLSX)
