# Supplementary material for: Cognitive ability, education and socioeconomic status in childhood and risk of post-stroke depression in later life: A systematic review and meta-analysis
Source: PLoS One. 2018 Jul 16;13(7):e0200525. doi: 10.1371/journal.pone.0200525 (PMC6047794; doi:10.1371/journal.pone.0200525)
Supplement: S1 File — (DOCX) [file pone.0200525.s002.docx]

**S1 File: All supporting information**

**S1 Appendix: Search Strategy**

MEDLINE Search Strategy

1. cerebrovascular disorders/ or basal ganglia cerebrovascular disease/ or exp brain ischemia/ or carotid artery diseases/ or carotid artery thrombosis/ or carotid stenosis/ or cerebral small vessel diseases/ or cerebral amyloid angiopathy, familial/ or stroke, lacunar/ or intracranial arterial diseases/ or cerebral arterial diseases/ or intracranial arteriosclerosis/ or exp "intracranial embolism and thrombosis"/ or exp stroke/ or leukoencephalopathies/ or leukoaraiosis/

2. exp *brain/ and *atrophy/

3. (isch?emi$ adj6 (stroke$ or apoplex$ or cerebral vasc$ or cerebrovasc$ or cva)).tw.

4. ((brain or cerebr$ or cerebell$ or vertebrobasil$ or hemispher$ or intracran$ or intracerebral or infratentorial or supratentorial or middle cerebral artery or MCA$ or anterior circulation or posterior circulation or basilar artery or vertebral artery or space-occupying) adj6 (isch?emi$ or infarct$ or thrombo$ or emboli$ or occlus$ or hypoxi$)).tw.

5. ((cerebell$ or vertebrobasil$ or hemispher$ or intracran$ or intracerebral or infratentorial or supratentorial or middle cerebr$ or MCA$ or anterior circulation or posterior circulation or basilar artery or vertebral artery or space-occupying) adj6 stroke$).tw.

6. ((brain or cereb$) adj6 (vascular or microvascular) adj6 (disease$ or disorder$)).tw.

7. (intracranial adj6 (disease$ or disorder$)).tw.

8. (cerebral adj6 (small vessel disease$ or microangiopath$ or amyloid angiopath$)).tw.

9. ((lacun$ or subcortical) adj6 (stroke$ or infarct$)).tw.

10. (leukoencephalopath$ or leukoaraiosis).tw.

11. (white matter adj6 (disease$ or hyperintensit$ or intensity$ or change$ or lesion$ or damage or abnormalit$ or integrity or tracts or infarct$ or structure)).tw.

12. ((brain or cerebral or intracranial) adj6 (arteriosclero$ or atherosclero$ or atrophy$ or microbleed$ or microhaem$ or microhem$)).tw.

13. exp *intracranial hemorrhages/ and (microbleed$ or microhaem$ or microhem$).tw.

14. or/1-13

15. cognition disorders/ or mild cognitive impairment/ or dementia/ or dementia, vascular/ or dementia, multi-infarct/

16. neurobehavioral manifestations/ or confusion/ or memory disorders/

17. mental processes/ or cognition/ or cognitive reserve/ or Arousal/ or Orientation/ or Attention/ or exp memory/ or perception/ or exp thinking/ or Awareness/ or Problem Solving/ or "Generalization (Psychology)"/ or "Transfer (Psychology)"/ or comprehension/ or Impulsive Behavior/ or Learning/

18. ((cogniti$ or arous$ or orientat$ or attention$ or concentrat$ or memor$ or recall or percept$ or think$ or sequenc$ or judg?ment$ or awareness or problem solving or generali?ation or transfer or comprehension or learning or mental process$ or (concept adj5 formation) or executive function$) adj6 (ability$ or function$ or difficult$ or impair$ or process$ or skill$ or performance or reserve or disorder$ or manifestation$ or declin$ or dysfunct$ or deficit$ or disabilit$ or problem$)).tw.

19. (cognition or confusion or dysexecutive syndrome$ or impulsive behavio?r$ or executive dysfunction$).tw.

20. or/15-19

21. exp intelligence/ or exp intelligence tests/

22. aptitude/ or aptitude tests/ or language tests/

23. education/ or educational status/ or exp educational measurement/

24. (intelligence or intelligent or IQ or intellectual or aptitude).tw.

25. (language adj6 (test$ or ability)).tw.

26. (education$ adj6 (status or attainment or measurement$)).tw.

27. ((mental or intellect$) adj6 (capacit$ or capabilit$ or abilit$ or performance)).tw.

28. or/21-27

29. child/ or child, preschool/ or adolescent/ or adult children/

30. (child$ or adolescen$ or youth or early life or early adult or pre-adult or early year$ or premorbid or pre-morbid).tw.

31. 29 or 30

32. 28 and 31

33. Education/ or Socioeconomic factors/ or educational status/ or educational measurement/ or psychology, educational/ or achievement/

34. (education or school$ or preschool or college or university or literate or literacy).tw.

35. ((educat$ or academi$ or schola$) adj6 (achieve$ or attain$ or level or qualification$ or performance or status)).tw.

36. or/33-35

37. socioeconomic factors/ or exp poverty/ or social class/ or social mobility/ or employment/ or unemployment/ or exp family characteristics/ or exp income/ or exp occupations/

38. ((soci$ or economic or living or family) adj6 (condition$ or factor$ or status or inequalit$ or standard$ or characteristic$ or size$ or wealth or position or depriv$ or income)).tw.

39. 37 or 38

40. child/ or child, preschool/ or adolescent/ or adult children/ or fathers/ or mothers/ or parents/

41. (child$ or adolescen$ or youth or early life or early adult or pre-adult or early year$).tw

42. 40 or 41

43. 39 and 42

44. ((father$ or mother$ or parent$) adj6 (income$ or occupation$ or job$)).tw

45. 43 or 44

46. 14 and 20 and 32 (CVD, cog and IQ))

47. 14 and 20 and 36 (CVD, cog and education)

48. 14 and 20 and 45 (CVD, cog and SES)

49. 14 and 32 (CVD and IQ)

50. 14 and 36 (CVD and education)

51. 14 and 45 (CVD and SES)

52. 46 or 47 or 48 (CVD, Cog and any early life factor)

53. 49 or 50 or 51 (CVD and any early life factor)

54. 52 or 53

NOTE: This search strategy was developed to identify studies examining early life factors (childhood/premorbid IQ, education and childhood SES) in relation to: a. Clinical and subclinical cerebrovascular disease b. Cognitive impairment in those with clinical and subclinical cerebrovascular disease c. Depression in those with clinical and subclinical cerebrovascular disease. In order to answer several questions screening was performed to identify studies which addressed any of these topics. Papers not relevant to the topic of the current work are excluded.

Table A: Details of included studies and extracted statistics used in the meta-analysis

| **Study** | **Setting** | **N** | **N with depression** | **N without depression** | **Age at follow up** | **Time post stroke** | **First stroke only** | **Measurement of stroke** | **Measure of early life factor** | **Measurement(s) of depression** | **Definition of depression** | **Previous depression excluded?** | **Results** | **p** |
| --- | --- | --- | --- | --- | --- | --- | --- | --- | --- | --- | --- | --- | --- | --- |
| **COGNITIVE ABILITY** | | | | | | | | | | | | | |  |
| Broadty (2007) | Hospital | 205 | 37 | 98 | Depression: 73.3 | 3 and/or 15 months | no | Clinical examination | Mean score on the NART | DSMIV | Major depression: DSMIV | no | Depression: 101.84 ±9.83 | 0.18 |
|  |  |  |  |  | No depression: 71.7 |  |  | Neuroimaging |  |  |  |  | No depression: 104.94 ±10.11 |  |
| **EDUCATION** | | | | | | | | | | | | | | |
| **Correlation** | | | | | | | | | | | | | | |
| Carod-Artal (2008) | Rehab hospital | 300 | 58 | 242 | 56.3 (14.3) | NS |  |  | Mean years | HADS | - |  | r= -0.25 | <0.001 |
| Schreiner (2001) | Outpatient clinic | 101 |  |  | Men- 54.5 Women- 69.9 | 45% ≤2 years |  |  | Duration in months | GDS- Short Form | - | yes | r= 0.105 | >0.05 |
| Spalletta (2002) | Hospital | 153 | RH- 49 | RH-38 | MDD- RH-64.1/ 63.2 | ≤1 year |  |  | Mean years | HADS | - |  | Right hem- r=-0.237 | 0.03 |
|  |  |  | LH- 39 | LH- 27 | MDD- LH- 67.8/69.7 |  |  |  |  | SCID | - |  | Left hem= r=0.087 | 0.49 |
| Visser (2014) | Outpatient clinic | 213 |  |  |  | ≥ 18 months |  |  | 7 levels ranging from 1 (< Primary school) to 7 (University degree) | CES-D | - | no | r= 0.028* (spearmans rho) |  |
| Donnellan (2016) | Hospital | 64 | 20 | 44 | 61 | ns | No | Clinical examination | Mean years | HADS | - | no | r= -0.34 (Pearsons) | ≤ 0.01 |
|  |  |  |  |  |  |  |  | Neuroimaging |  |  | - |  |  |  |
| **Mean years** | | | | | | | | | | | | | | |
| Brodaty (2007) | Hospital | 205 | 37 | 98 | Depression: 73.3 | 3 and/or 15 months |  | Clinical examination | Mean years | DSMIV | Major depression: DSMIV | no | Depression: 10.39±2.65 | 0.52 |
|  |  |  |  |  | No depression: 71.7 |  | No | Neuroimaging |  |  |  |  | No depression: 10.03±2.91 |  |
| Choi-Kuron (2012) | Outpatient clinic | 469 | 83 | 386 | Depression: 63.2 | 3 months | No | Neuroimaging | Mean years | BDI | PSD: BDI >13 or | yes | Depression: 8.8 ± 4.9 | <0.05 |
|  |  |  |  |  | No depression: 61.4 |  |  |  |  | DSMIV | Major depression: DSMIV |  | No depression: 10.1 ± 5 |  |
| Kim (2000) | Outpatient clinic | 149 | 27 | 121 | 40-80 | 2-4 months | Yes | Clinical examination | Mean years | DSMIV | Major depression: DSMIV | yes | Depression: 10.5±4.7 |  |
|  |  |  |  |  | 62 |  |  | Neuroimaging |  | BDI |  |  | No depression: 10.6 ± 4.7 |  |
| Snaphaan (2009) | Hospital | 283 | 43 | 241 | Depression: 64.9 | ns | No | Clinical examination | Median years | HADS | Depressive symptoms >8 | no | Depression: 4(1-7) | <0.01 |
|  |  |  |  |  | No depression: 65.9 |  |  | Neuroimaging |  |  |  |  | No depression): 5 (1-7) |  |
| Starkstein (1993) | Hospital | 80 | 18 | 44 | Depression:51.0 | 10 days | Yes | Clinical examination | Mean years | DSM-III | Major depression: DSM-III | no | Depression: 9.9±2.9 |  |
|  |  |  |  |  | No depression: 61.1 |  |  | Neuroimaging |  | PSE |  |  | No depression: 9.5±3.1 |  |
| Tang (2005) | Hospital | 189 | 27 | 158 | Depression: 67.3 | 3 months | No | Clinical examination | Mean years | SCID-DSM-IV | Major or minor depression: SCID-DSM-IV | no | Depression: 3.5±3.3 | 0.04 |
|  |  |  |  |  | No depression: 68.3 |  |  | Neuroimaging |  |  |  |  | No depression:5.5±4.8 |  |
| Tateno (2002) | Hospital | 354 | 73 | 281 | Major depression: 58.2 | ns | No | Neuroimaging | Mean years | DSMIV  HADS | Major depression: DSMIV | no | Major depression: 10±3.3 |  |
|  |  |  |  |  | Minor or no depression: 61.7 |  |  |  |  |  |  |  | Minor or no depression: 10.1 ±3.9 |  |
| Tene (2016) | Hospital | 306 | 45 | 261 | Depressive episode: 68.2 | 72 hours | No | Neuroimaging | Mean years | GDS | Depressive episode: GDS ≥6 | no | Depressive episode: 11.8±3.2 | <0.05 |
|  |  |  |  |  | No depression: 66.9 |  |  |  |  |  |  |  | No depression: 13.4±3.7 |  |
| **Frequencies** | | | | | | | | | | | | | | |
| Berg (2001) | Outpatient clinic | 100 | 24 | 65 | 55.2 | 2 weeks | Yes | Clinical examination | ≤ 8 years | BDI | Mild depression: BDI ≥10 | no | ≤ 8 years: 9 vs 37 | 0.10 |
|  |  |  |  |  |  |  |  | Neuroimaging | >8 years | DSMIV |  |  | >8 years: 15 vs 28 |  |
| Fayoye (2009) | Hospital | 118 | 47 | 71 | ns | Mean: 11 months | No | ns | None  Primary school  Secondary school  Tertiary | BDI | Mild depression: BDI ≥10 | no | <Secondary school: 34 vs 26  ≥Secondary school: 13 vs 45 | 0.001 |
| Hirata (2016) | Population | 546 | 87 | 459 | Depression: 55.8  No depression: 65.8 | Mixed | No | Self-report | < High school  High school  > High school | PHQ-8 | Moderate depression: ≥10 | no | <HS: 28 vs 134  ≥HS: 59 vs 325 |  |
| Jiang (2014) | Hospital | 329 | 98 | 231 | Depressive symptoms: 69.2  No depression: 66.6 | 2-6 weeks | No | Neuroimaging | Illiteracy  Elementary school  High school  > High school | MADRAS  DSMIV | Depressive symptoms: MADRAS ≥ 7 | no | ≤ High school: 73 vs 150  ≥ High school: 25 vs 81 |  |
| Nys (2006) | Hospital | 91 | 48 | 43 | Depression: 62.3  No depression: 60.8 | 6-10 months | Yes | Clinical examination  Neuroimaging | 7 levels ranging from 1 (< Primary school) to 7 (University degree) | MADRAS | Depressive symptoms: MADRAS ≥7 | yes | High education: 16 vs 22  Low education: 32 vs 21 | 0.09 |
|  |  |  |  |  |  |  |  |  | Dichotomised at the median |  |  |  |  |  |
| Paolucci (2006) | Outpatient clinic | 1,064 | 383 | 681 | Depression: 67.4 | Between 2-6 weeks | No | Neuroimaging | ≤ 8 years | DSMIV | Major depression: DSMIV | no | ≤ 8 years: 86 vs 148 |  |
|  |  |  |  |  |  |  |  |  |  | BDI |  |  |  |  |
|  |  |  |  |  | No depression: 67.1 |  |  |  | > 8 years | Visual Analogue Dysphoria Scale | sad face' |  | >8 years: 297 vs 533 |  |
| Shi (2015) | Hospital | 757 | 201 | 344 | Early onset depression: 61.25 | 2 weeks-1 year | Yes | Neuroimaging | <High school | DSMIV | Major depression: DSMIV | no | <HS: 125 vs 220 |  |
|  |  |  |  |  | Late onset depression: 60.21 |  |  |  | ≥High school |  |  |  | ≥HS: 76 vs 124 |  |
|  |  |  |  |  | No depression: 61.07 |  |  |  |  |  |  |  |  |  |
| Vataja (2001) | Hospital | 275 | 109 | 166 | Depression: 70.3 | 3-4 months | No | Clinical examination | ≤ 6 years | DSM-III-R | Major depression: DSM-III-R | no | ≤ 6 years: 32 vs 51 | 0.56 |
|  |  |  |  |  | No depression: 70.9 |  |  | Neuroimaging | >6 years | PSE (10th edition) |  |  | >6 years: 77 vs 115 |  |
| Verdelho (2004) | Hospital | 108 | 46 | 62 | Depression: 69.5 | 6 months | No | Neuroimaging | ≤ Primary school | MADRAS | Depressive symptoms: MADRAS ≥7 | no | ≤ Primary school: 38 vs 51 |  |
|  |  |  |  |  | No depression: 71 |  |  |  | >Primary school | CAMDEX |  |  | >Primary school: 8 vs 11 |  |
| Yu-Mei (2002) | Hospital | 126 | 76 | 50 | Depression: 67.5 | ns | No | Clinical examination | ≤ 6 years | HDRS | HDRS ≥8 (mild depression) | no | ≤ 6 years: 39 vs 13 | <0..01 |
|  |  |  |  |  | No depression: 69.8 |  |  | Neuroimaging | >6 years | Chinese classification of mental disorders and diagnostic criteria 2nd edition |  |  | >6 years: 37 vs 37 |  |
| Zhang (2009) | Community | 276 | 180 | 96 | 67.5 | Mean: 13 months | No | Clinical examination  Neuroimaging | Illiteracy  Primary school  Middle school (8 years)  >Middle school (>8 years) | Self-Rating Depression scale | mild depression | no | ≤ Middle school: 147 vs 223  >Middle school: 33 vs 51 |  |
| Zhang (2005) | Hospital | 312 | 135 | 177 | ns | within 48 hours | No | Clinical examination  Neuroimaging | Elementary school  Middle school  Junior college | HDRS | HDRS ≥8 (mild depression) | no | ≤ Middle school: 86 vs 129  >Middle school: 49 vs 48 |  |
| **Odds ratio** | | | | | | | | | | | | | | |
| Alteri (2012) | Hospital | 105 | 43 | 62 | 64.4 | 1 month | No | ns | < 8 years  ≥ 8 years | DSMIV | Major depression: DSM-IV | yes | OR: < 8 years vs ≥ 8 years: 1.6 (1.023-2.49)† | 0.04 |
| Naess (2005) | Population | NS | 196 | NS | 15-49 | Mean time: 6 years | Yes | Clinical examination  Neuroimaging  Centralised health statistics | Low/high education  Not defined | MADRAS | Mild depression : MADRAS ≥7 | no | OR: 1.33 (0.66-2.70)† | 0.43 |
| Paul (2013) | Population | 241 | 113 | 128 | Depression: 64.7  No depression: 60.8 | Various | No | Clinical examination  Self-report | Mean years | GDS (Bengali version) | Severe depression: GDS ≥21 | no | OR: For every year decrease in education:  1.09 (1.02-1.17) §† | 0.01 |
| Paolucci (1999) | Hospital | 470 | 129 | 341 | 66.26 | Mean: 44.3 days | Yes | Clinical examination | ≤8 years | HDRS | Severe depression: HDRS ≥18 | yes | OR: <High school: 0.62 (0.40-0.96)≠† | 0.03 |
|  |  |  |  |  |  |  |  | Neuroimaging  Case note review | > 8 years | Visual Analogue Dysphoria Scale | Clinical picture of depressed mood at interview |  |  |  |
| Schepers (2009) | Hospital | 131 | 33 | 98 | 56.3 | 1 year | Yes | ns | <University degree  Higher professional or University degree | CES-D | Depressive episode: ≥16 | no | OR: <University degree: 0.90 (0.36-2.26) † | 0.83 |
| Sienkiewicz (2010) | Hospital | 242 | 82 | 160 | Depression: 66.1  No depression: 65.2 | 3 months | Yes | Clinical examination  Neuroimaging | ≤ Primary school  >Primary school | GDS | Depressive symptoms: GDS >5 | no | OR: <Secondary school vs ≥Secondary school: 2.1 (1.2-3.8) | 0.01 |
| Tang (2011) | Hospital | 235 | 84 | 151 | Depression: 67.1 | 3 months | No | Clinical examination | Years | GDS | Depressive symptoms: GDS≥7 | yes | OR For every decrease in education: 1.04 (0.98-1.10) ¤† | 0.25 |
|  |  |  |  |  | No depression: 65.9 |  |  | Neuroimaging |  |  |  |  |  |  |
| Van de Port (2007) | Hospital | 165 | 31 | 134 | 57 | 3 years | Yes | Neuroimaging | <University education  University education | CES-D | Depressive episode: CED-S ≥16 | no | <University education: 1.56 (0.56-4.32) † | 0.39 |

* adjusted for age, sex, income, smoking, age, cognitive dysfunction and activities of daily living

≠ adjusted for age, sex

§ adjusted for age, sex, income, smoking, age, cognitive dysfunction and activities of daily living

¤ adjusted for sex, lobar cerebral micro-bleeds, Lubben Social Network Scale score, Mini-Mental State Exam score, diabetes, National Institute of Health Stroke Scale score

† inverted for meta-analysis to demonstrate inverse relationship

DSM: Diagnostic Statistical Manual; BDI: Beck’s Depression Inventory; HADS: Hospital Anxiety and Depression Scale; PSE: Present State Exam; HDRS: Hamilton Depression Rating Scale; GDS: Geriatric Depression Scale; PHQ: Patient Health Questionnaire; MADRAS: Montgomery-Asberg Depression Rating Scale; CES-D: Centre for Epidemiologic Studies Depression Scale; CAMDEX: The Cambridge Examination for Mental Disorders of the Elderly.

Figure A: Frequencies of scores on the subscale of the quality assessment for all included studies.


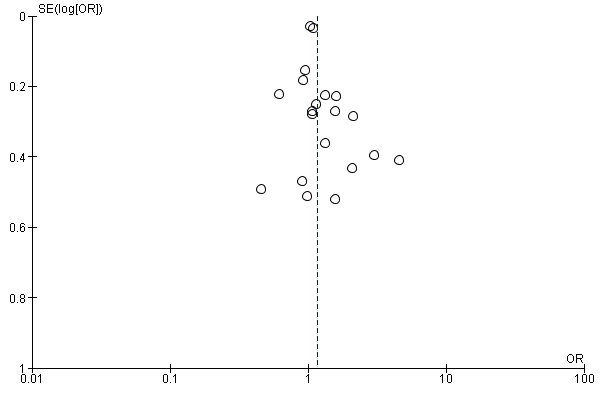


Figure B: Funnel plot of studies examining education and post-stroke depression

**Figures C-J: Sensitivity analysis for studies examining education level and post-stroke depression.**


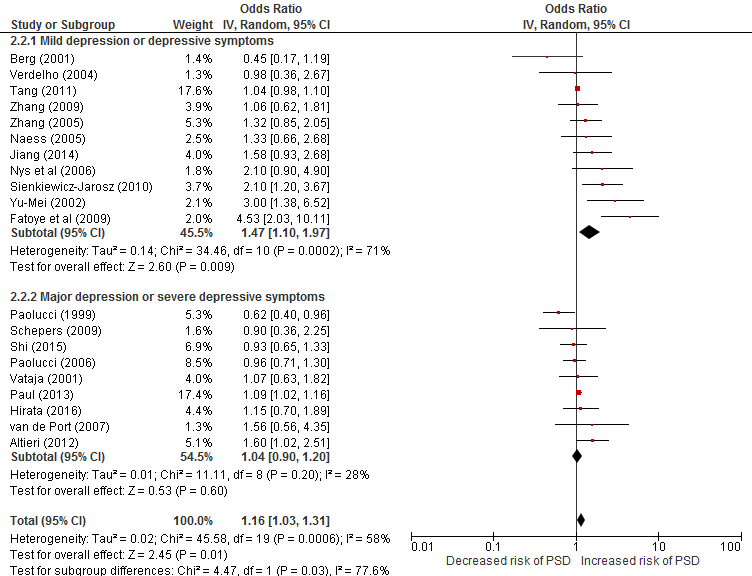


Low education

Figure C: Sensitivity analysis comparing post-stroke depression measurement: Mild depression or depressive symptoms vs major depression or severe depressive symptoms by education level; OR>1=low education increases risk of post-stroke depression.


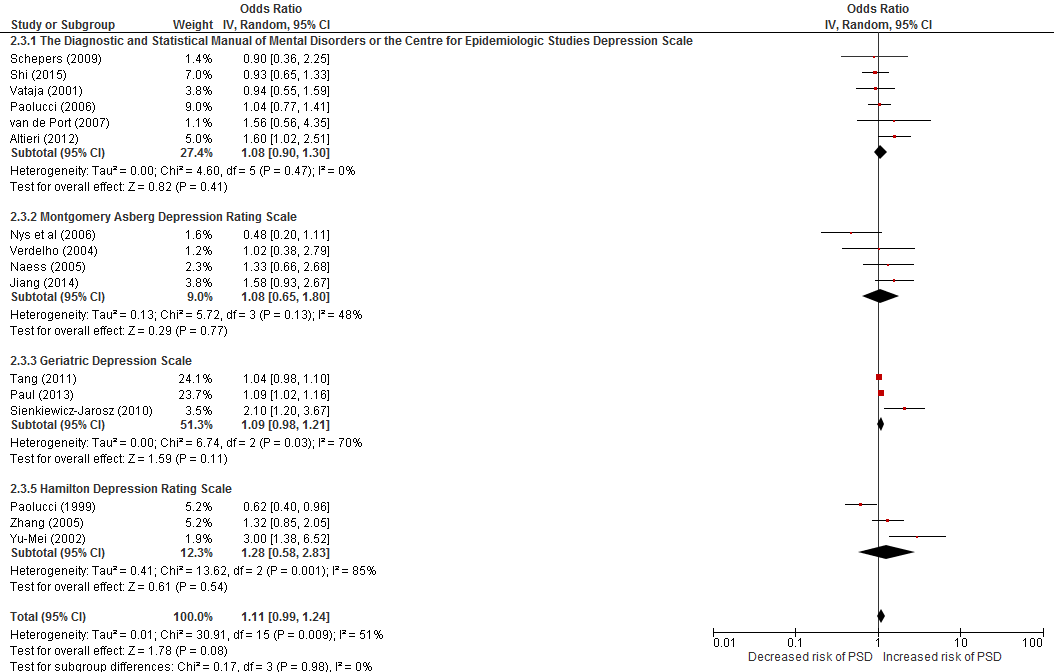


Low education

Figure D: Sensitivity analysis comparing studies according to the depression scale used by education level and risk of post-stroke depression; OR>1=low education increases risk of post-stroke depression.


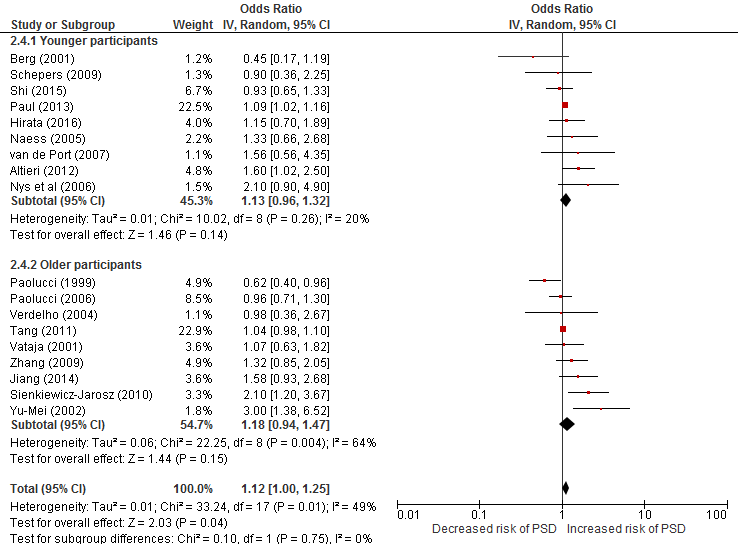


Low education

Figure E: Sensitivity analysis comparing studies that included younger (mean age <65 years) vs those that included older (mean age >=65 years) participants by education level and risk of post-stroke depression; OR>1=low education increases risk of post-stroke depression.


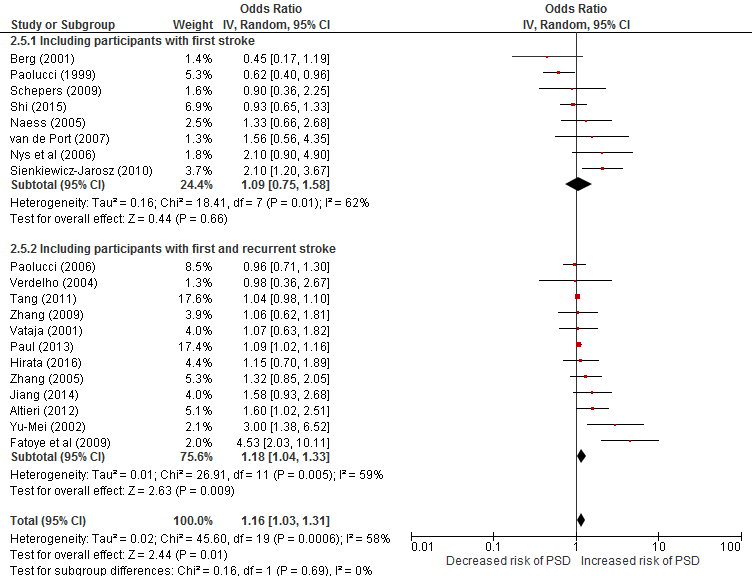


Low education

Figure F: Sensitivity analysis comparing studies with participants with first stroke vs studies with participants with first and recurrent stroke by education level and risk of post-stroke depression; OR>1=low education increases risk of post-stroke depression.


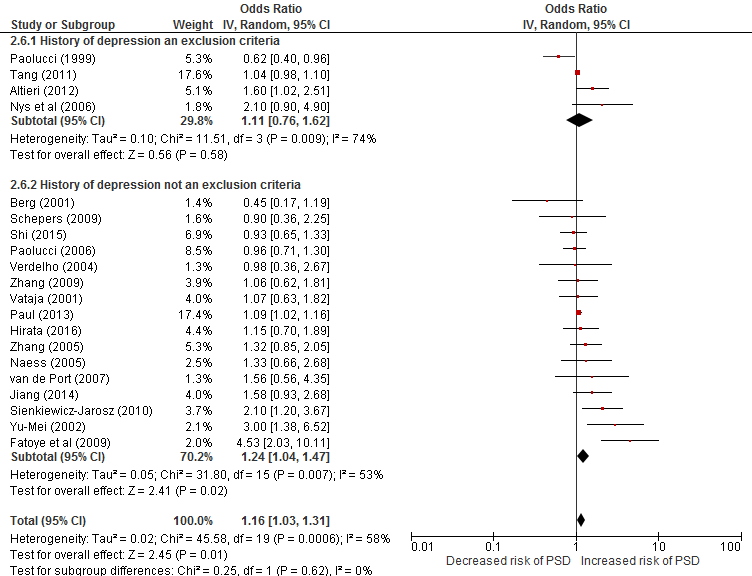


Low education

Figure G: Sensitivity analysis comparing studies with participants with a history of depression as an exclusion criteria vs studies without history of depression as an exclusion criteria by education level and risk of post-stroke depression; OR>1=low education increases risk of post-stroke depression.


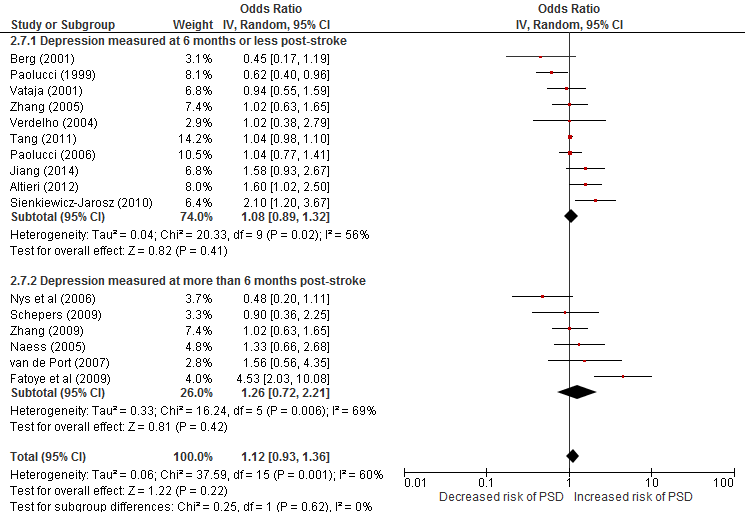


Low education

Figure H: Sensitivity analysis comparing studies with depression measured at 6 months or less vs studies with depression measured at more than 6 months post stroke by education level and risk of post-stroke depression; OR>1=low education increases risk of post-stroke depression.


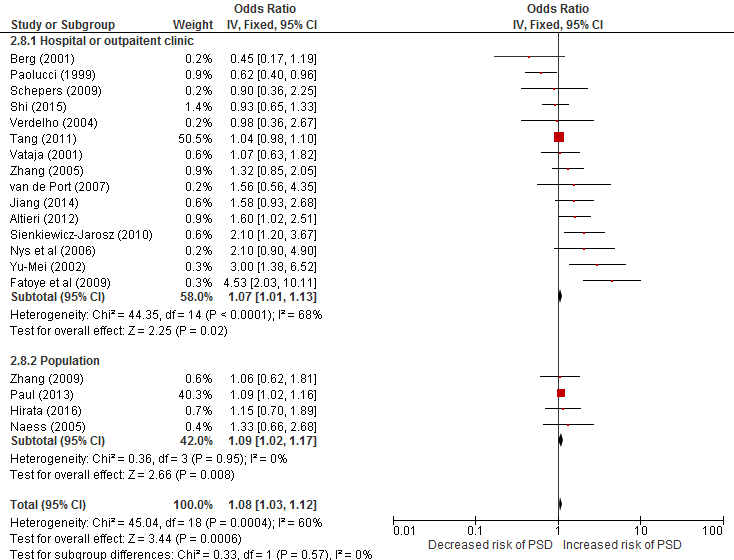


Low education

Figure I: Sensitivity analysis comparing population cohort studies vs hospital/outpatient studies by education level and risk of post-stroke depression; OR>1=low education increases risk of post-stroke depression.


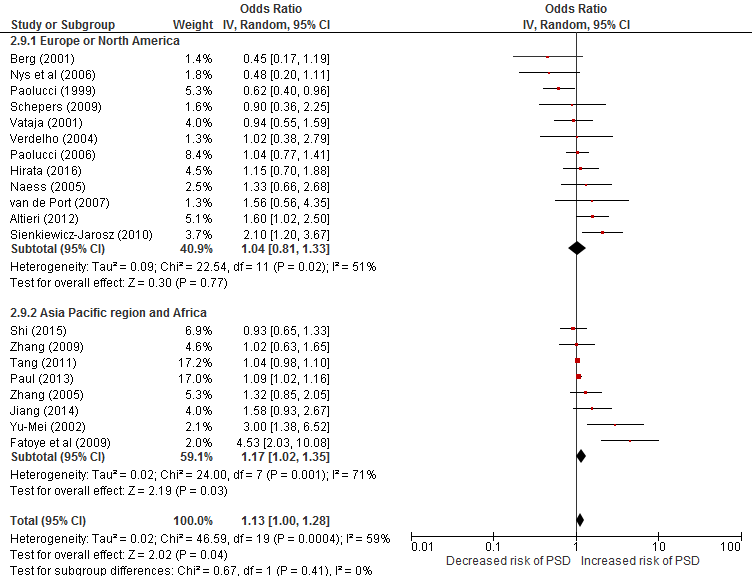


Low education

Figure J: Sensitivity analysis comparing studies conducted in Europe or North America vs the Asia Pacific region or Africa by education level and risk of PSD; OR>1=low education increases risk of stroke.

Low education
